# Impact of aging on CD146^+^ mesenchymal stromal cells-mediated regulation of bone marrow CD34^+^ hematopoietic stem/progenitor cell mobilization

**DOI:** 10.3389/fbioe.2026.1802093

**Published:** 2026-05-07

**Authors:** Marzia Campanile, Miruna Larisa Naie, Milena Bertolotti, Ivan Muradore, Chiara Riccardi, Andrea Rampin, Giuseppe Noto, Paolo Angelo Laviano, Viola Melone, Domenico Palumbo, Roberta Tarallo, Alessandro Fantin, Elena Butoi, Gaia Spinetti

**Affiliations:** 1 Laboratory of Cardiovascular Pathophysiology and Regenerative Medicine, IRCCS MultiMedica, Milan, Italy; 2 Department of Biosciences, University of Milan, Milan, Italy; 3 Inflammation Department, Institute of Cellular Biology and Pathology “Nicolae Simionescu”, Bucharest, Romania; 4 Orthopedic Unit, IRCCS MultiMedica, Milan, Italy; 5 Laboratory of Molecular Medicine and Genomics, Department of Medicine, Surgery and Dentistry, University of Salerno, Baronissi, Italy

**Keywords:** aging, bone marrow, hematopoietic stem/progenitor cells, *In vitro* models, migration, stromal cells, vasculature

## Abstract

**Background:**

The study of age-associated changes in the bone marrow (BM), a key organ for hematopoiesis and immune regulation, is crucial to understanding inflammatory processes linked to cardiovascular diseases. Notably, aging is associated with impaired mobilization of BM-derived cardioprotective CD34^+^ hematopoietic stem/progenitor cells (HSPCs), resulting in a lower frequency in the circulation and poorer cardiovascular outcomes. HSPCs pharmacological mobilizers (G-CSF, AMD3100) and sympathetic signaling stimulator (norepinephrine, NE) function mainly by acting on CD146^+^ Bone marrow stromal cells (BMSCs), key cellular players in this process. In this study, we developed a human *in vitro* BM simplified experimental model to assess the impact of aging on CD146^+^ BMSCs in this context.

**Methods:**

CD146^+^ BMSCs were isolated from 29 human subjects, stratified into middle-aged (middle-aged, ≤66 years; N = 13) and older adults (older adult, ≥75 years; N = 16). For functional assays, bone marrow HSPCs from a single donor (female, 64 years) were used. The *in vitro* BM simplified experimental model was developed by co-culturing CD146^+^ BMSC and CD34^+^ HSPCs in a transwell system. After treatment with mobilizing agents, CD146^+^ BMSCs’ viability, HSPC migration, transcriptome, metabolism, and paracrine activity were assessed.

**Results:**

AMD3100 enhanced HSPC migration in the model using MA-derived CD146^+^ BMSCs but not in OA-derived cells. RNA sequencing identified 9 age-associated genes, with validated downregulation of *NRK* and upregulation of *PDK4*, *AQP1*, and *LMO2* in OA CD146^+^ BMSC. Moreover, a differential gene expression response to mobilizing treatments was observed between groups. Cell-conditioned media from OA CD146^+^ BMSCs showed stronger chemoattractant effects on peripheral blood mononuclear cells and presented increased VCAM-1 levels. No age-related effects on oxidative respiration were observed.

**Conclusion:**

In this study, a BM *in vitro* co-culture system was developed to study CD146^+^ BMSC-dependent CD34^+^ mobilization in a subject-specific manner, and the complexity of the impact of aging on CD146^+^ BMSC and their response to mobilizing agents was highlighted.

## 1 Introduction

Age-related remodeling of the bone marrow (BM) niche has emerged as a critical determinant of systemic inflammation and cardiovascular vulnerability (Mangialardi et al., 2014; Bonora et al., 2022; Kohler et al., 2009; Aranda et al., 2025).

As the primary reservoir of hematopoietic and immune progenitors, the BM contributes to cardiovascular homeostasis through the regulated retention and mobilization of CD34^+^ hematopoietic stem and progenitor cells (HSPCs). In older individuals, a marked reduction in circulating CD34^+^ cells has been associated with adverse cardiovascular outcomes, fueling interest in pharmacological strategies aimed at enhancing progenitor mobilization (Moresi et al., 2005; Shimizu et al., 2020; Cohen et al., 2013; Patel et al., 2015; Albiero and Fadini, 2018). Consistently, aged mice-derived early hematopoietic stem cells localize more distantly to the endosteum compartment than cells derived from young animals, although showing reduced adhesion to stromal cells, suggesting that aging alters their spatial organization within the BM niche (Kohler et al., 2009).

Self-renewal, proliferation, migration, and differentiation of HSPCs into distinct lineages are tightly regulated to preserve homeostasis under steady-state and stress conditions. These coordinated cell fate decisions depend not only on cell-intrinsic mechanisms, including transcriptional, epigenetic, and metabolic programs, but also on cell-extrinsic signals provided by the BM microenvironment (Mosteo et al., 2021; Ferland-McCollough et al., 2018; Spinetti et al., 2013; Dang et al., 2015).

At the molecular level, CD34^+^ cell mobilization is governed by the balance between chemotactic retention signals and adhesive interactions within the niche. Reduced levels of BM CXCL12, also known as stromal-derived factor 1α, SDF-1α, represent a major driver of CD34^+^ cells egress, whereas stable anchorage to stromal and vascular components is mediated by cell adhesion molecules (CAMs). Accordingly, proteases-mediated disruption of these adhesive interactions constitutes a critical step in mobilization. In parallel, intracellular redox balance has emerged as an additional regulatory layer, with increased reactive oxygen species (ROS) levels promoting CD34^+^ HSPC mobilization through modulation of signaling pathways involved in adhesion, migration, and niche responsiveness (Albiero and Fadini, 2018; Itkin et al., 2016; Chang et al., 2022).

Pharmacological mobilization of CD34^+^ cells is a crucial process in BM transplantation clinical medicine, and is being explored as a therapeutic strategy to enhance recovery in cardiovascular diseases (Albiero and Fadini, 2018). Among mobilizing agents, the most widely used is the granulocyte colony-stimulating factor (G-CSF), which induces mobilization indirectly by promoting extensive remodeling of stromal and vascular compartments, leading to reduced CXCL12 availability and alterations in extracellular matrix composition (Albiero and Fadini, 2018; Chang et al., 2022; Day et al., 2015). In contrast, the CXCR4 antagonist AMD3100 (plerixafor) acts directly on the CXCR4/CXCL12 axis, rapidly disrupting HSPC retention within the niche. In addition, sympathetic nervous system signaling, mediated by norepinephrine (NE), also reduces CXCL12 BM levels. Despite the clinical relevance of these pathways, it remains unclear whether age-associated defects in mobilization originate from intrinsic alterations in HSPCs or from impaired responsiveness of the BM niche (Albiero and Fadini, 2018; Lucas et al., 2012).

Bone marrow stromal cells (BMSCs) are key regulators of CD34^+^ HSPC migration through CXCL12 secretion and by shaping the molecular and structural properties of the BM vascular niche. Within the heterogeneous BMSC compartment, CD146^+^ BMSCs, which identify pericytes, constitute a perivascular subset functionally integrated into the vascular niche and crucial for CD34^+^ HSPC regulation (Bowles et al., 2020; Tormin et al., 2011; Espagnolle et al., 2014; Harkness et al., 2016; Walenda et al., 2010; Crisan et al., 2008). However, the specific contribution of primary human CD146^+^ BMSCs to CD34^+^ cells mobilization, particularly in an age-dependent context, has not been directly investigated in controlled human *in vitro* systems.

Until recently, studies of the BM niche have relied largely on murine models, with inherent limitations in translating findings to human physiology (Mosteo et al., 2021). Advances in biomaterials and bioengineering have enabled the reconstruction of selected elements of the human BM microenvironment *in vitro*; however, faithfully recapitulating the architecture, cell composition, cell-cell interactions, structural differences, and the composition of the extracellular matrix, as well as the availability of extrinsic molecular cues from growth factors and cytokines of the BM vascular niche, remains challenging (Busch et al., 2024). Existing *in vitro* models have primarily focused on CD34^+^ HSPC engraftment, proliferation, or malignant cell homing. When CD34^+^ cell migration has been addressed, BMSCs were rarely included; instead, endothelial cells were more commonly employed (Campanile et al., 2023; de Barros et al., 2010; Mohle et al., 1997; Aleman et al., 2019; Voermans et al., 2001; Kessinger et al., 2005; Kim and Broxmeyer, 1998; van Buul et al., 2002; Mohle et al., 1999; Aiuti et al., 1997). Thus, the molecular determinants governing direct interactions between primary human CD146^+^ BMSCs and CD34^+^ HSPCs during directed migration in response to mobilizing agents remain only partially understood.

Here, we developed a human *in vitro* BM simplified experimental model based on primary CD146^+^ BMSCs to directly interrogate age-dependent and niche-specific regulation of CD34^+^ HSPC migration in response to clinically relevant mobilizing stimuli, with implications for personalized regenerative strategies.

## 2 Materials and methods

### 2.1 Primary human BM cell isolation and treatment

BM cells were isolated from femoral heads, otherwise discarded as surgical waste, obtained upon written informed consent from subjects undergoing hip replacement surgery due to coxarthrosis. The applied exclusion criteria included participation in other clinical trials within the 3 months prior to signing informed consent; recent acute myocardial infarction or stroke; clinical conditions associated with a life expectancy of less than 2 years; drug or alcohol abuse; any other condition that could impair adherence to the study; and failure to provide written informed consent. Subjects were stratified into two groups based on age: middle-aged adults, MA (56 ± 6.16 years old), and older adults, OA (79.88 ± 3.85 years old). Subjects’ characteristics and sample applications are summarized in Table 1. All procedures were conducted in accordance with the Declaration of Helsinki and approved by the local Ethics Committee (protocol n. 540/2022). Femoral heads were excavated to collect BM pulp, which was extensively washed, filtered through a 100-µm mesh, and subjected to density gradient separation using Histopaque-1077 (Sigma-Aldrich, cat # 10771). Mononuclear cells (MNCs) were seeded at a density of 1 × 10^5^- 1 × 10^6^ cells, depending on cell viability, and cultured for 7 days in complete medium that means vascular cell basal medium (ATCC, cat # PCS-100–030 VCBM) supplemented with endothelial cell growth kit (ATCC, cat # ECGK-VEGF PCS-100-041), heat inactivated fetal bovine serum (Gibco, cat # A5256701) for a final concentration of 20%, penicillin-streptomycin (Euroclone, cat # ECB3001D) to a final concentration of 100 U/mL and 100 μg/mL, respectively. Cells were then expanded, and upon reaching confluence, CD146^+^ BMSCs were isolated using a CD146^+^ magnetic bead positive selection (Miltenyi Biotec, cat # 130-093–596) and cryopreserved in a FBS-10% DMSO solution. BM CD34^+^ HSPCs were isolated from one subject (female, 64 years old) using the Diamond CD34^+^ Magnetic Bead Isolation Kit (Miltenyi Biotec, cat # 130-094–531) according to the manufacturer’s instructions. Briefly, lineage-negative cells were first obtained from mononuclear cells by negative selection, followed by positive selection to isolate CD34^+^ cells which were subsequently cryopreserved. The BM cells isolation protocol is depicted in Supplementary Figure S1A.

**TABLE 1:** Study subject characteristics and experimental usage”. Legend: “Table showing study subject characteristics (age and sex) and the experimental procedures in which each subject was included. Subjects are identified using coded IDs (MA or OA series), with distinct coding for the two groups analyzed. Middle-aged adult, MA and older adult, OA.

Sample	Sex	Age (years old)	MTT	qPCR	RNA-seq	CCM	Seahorse/migration
MA1	M	46	–	✓	✓	–	✓
MA2	M	52	✓	✓	–	✓	✓
MA3	M	47	✓	✓	–	✓	✓
MA4	M	55	✓	✓	–	✓	–
MA5	M	56	✓	✓	–	–	–
MA6	M	57	–	–	✓	–	–
MA7	M	51	✓	✓	–	–	–
MA8	F	60	✓	✓	✓	–	–
MA9	F	65	✓	✓	✓	–	–
MA10	F	56	✓	✓	✓	–	–
MA11	F	64	✓	✓	–	✓	–
MA12	F	53	✓	✓	–	–	–
MA13	F	66	–	–	–	✓	–
OA1	M	75	✓	✓	–	✓	✓
OA2	M	78	✓	✓	✓	–	✓
OA3	M	76	✓	–	–	✓	✓
OA4	M	84	–	✓	–	✓	–
OA5	M	87	–	✓	✓	–	–
OA6	M	77	–	✓	–	–	–
OA7	F	84	–	–	–	✓	–
OA8	F	75	–	–	–	–	–
OA9	F	76	✓	✓	–	–	–
OA10	F	77	✓	✓	–	–	–
OA11	F	78	✓	–	–	–	–
OA12	F	80	–	✓	✓	–	–
OA13	F	80	✓	✓	–	✓	–
OA14	F	82	✓	✓	✓	–	–
OA15	F	83	✓	✓	–	–	–
OA16	F	86	✓	✓	✓	–	–

✓ = assay performed; – = not performed.

### 2.2 BM cell culture and treatments

Passage 1 CD146^+^ BMSCs were thawed in FBS, centrifuged, and seeded in complete medium at a density ranging from 5 × 10^3^ to 5 × 10^4^ cells/cm^2^, depending on cell viability. Cells were cultured until reaching confluency and were used for experiments exclusively at passage 3.

Bone marrow-derived CD34^+^ HSPCs were thawed and expanded in StemMACS™ HSC Expansion Medium XF, human (Miltenyi Biotec, cat. no. 130-100–473), supplemented with StemMACS™ HSC Expansion Cocktail, human (Miltenyi Biotec, cat. no. 130-100–843). Cells were expanded for 2 weeks, and cell density was maintained between 5 × 10^5^ and 5 × 10^6^ cells/mL.

Where indicated, CD146^+^ BMSCs were treated in complete medium with 100 µM AMD3100 (Tocris, cat # 3299), 10 ng/mL G-CSF (Sigma-Aldrich, cat # SRP3263), or 500 ng/mL norepinephrine (NE; Sigma-Aldrich, cat # A7257) for 48 h. In the control condition, cells were cultured in complete medium alone.

### 2.3 Flow cytometry

The identity of isolated CD146^+^ BMSCs and CD34^+^ HSPCs was assessed by flow cytometry. For CD146^+^ BMSC analysis, cells were stained at the passage used for the experiments. Briefly, cryopreserved cells were thawed at passage 2, expanded, detached, and stained at passage 3; a total of 0.5 × 10^5^ cells were analyzed. For CD34^+^ HSPC staining, 1 × 10^5^ cells were used. Samples were resuspended in 50 µL of MACS buffer (PBS containing 0.5% BSA and 2 mM EDTA) and stained with antibodies (1:50 working dilution) for 20 min at room temperature. Cells were then washed once with 1 mL of MACS buffer, resuspended in 0.5 mL of MACS buffer, and analyzed by flow cytometry using a BD LSRFortessa™ X-20 Cell Analyzer with the appropriate laser/detector configuration. The following antibodies were used: PDGFRB/CD140b-APC-Vio770 (Miltenyi, cat # 130-129–294), CD146-PE-Vio615 (Miltenyi, cat # 130-125–731), CD45-VioBright B515 (Miltenyi, cat # 130-110–640), CD90-PE-Vio770 (Miltenyi, cat # 130-114–862), CD31-VioBlue (Miltenyi, cat # 130-110–674), and CD34-PE (Miltenyi, cat # 130-120–515). For CD146^+^ BMSC characterization, all listed antibodies were used. CD34^+^ HSPCs were identified based on CD45 and CD34 expression, and cell viability was assessed using Zombie NIR™ Fixable Viability Kit (BioLegend, cat # 423106). Approximately 71% of expanded CD34^+^ HSPCs displayed a CD45^dim^ CD34^+^ immunophenotype, confirming effective enrichment of the BM-HSPC population (Supplementary Figure S1B, pink-highlighted sub-panel). CD146^+^ BMSC identity was confirmed by the absence of the leukocyte marker CD45 (0.024% ± 0.026) and of the endothelial markers CD31 (0.26% ± 0.36) and CD34 (0.43% ± 1.03), together with the presence of the mesenchymal marker CD90 (82.58% ± 5.79) and of the pericyte-associated markers CD146 (79.12% ± 8.98) and PDGFRβ (94.72% ± 2.56) across N = 3 samples (Supplementary Figure S1B, yellow-highlighted sub-panels).

### 2.4 Immunofluorescence analysis

CD146^+^ BMSCs were further characterized by immunofluorescence analysis. At passage 3, CD146^+^ BMSCs were seeded at a density of 5 × 10^3^ cells/cm^2^ in µ-Slide VI (IBIDI, cat # 980606) and fixed after 48 h with 4% paraformaldehyde for 15 min at room temperature. Cells were then washed three times with PBS and incubated for 1 h in PBS containing 0.1% Tween-20% and 5% goat serum for blocking and permeabilization. Subsequently, cells were incubated overnight at 4 °C with the following primary antibodies (1:100 working dilution): PDGFR-β (Abcam, cat # ab154556, rabbit), CD146 (Thermo Fisher Scientific, cat # MA5-29414, rabbit), and NG2 (Abcam, cat # ab83508, mouse). NG2 was co-stained with each of the other primary antibodies. After three washes with PBS-T, cells were incubated for 2 h at room temperature with 1:500 secondary antibodies anti-mouse Alexa Fluor™ 488 (Thermo Fisher Scientific, cat # A-11001) and anti-rabbit Alexa Fluor™ 555 (Thermo Fisher Scientific, cat # A-21428), washed, and counterstained with DAPI (Sigma-Aldrich, cat #D9542).

### 2.5 Cells employed in functional assays

EA.hy926 cells (ATCC, cat # CRL-2922), a human-endothelial-derived cell line, were cultured in DMEM supplemented with fetal bovine serum 10%, penicillin (100 U/mL), and streptomycin (100 μg/mL), in a humidified incubator with 5% CO_2_.

Peripheral blood mononuclear cells (PBMCs) were isolated from fresh human peripheral blood (protocol n. 5451, 15/09/2023) by density gradient centrifugation using Ficoll-Paque PLUS (or Histopaque-1077). Blood was diluted 1:1 with PBS and layered over the density medium, followed by centrifugation at 400 *g* for 30 min at room temperature without brake. The mononuclear cell layer was collected, washed twice with PBS, and resuspended for subsequent analyses.

### 2.6 Cell-conditioned medium collection

CD146^+^ BMSCs from subjects of different age were seeded in 12-well plates at a density of 1.5 × 10^4^ cells/cm^2^. After 24 h of culture, cells were treated with AMD3100, G-CSF, NE, or complete medium alone. Cells were then washed with PBS and incubated with 800 µL of vascular basal medium. After 24 h, the medium was collected, centrifuged at 1.000 *g* for 10 min, and the supernatant was harvested as cell-conditioned medium (CCM).

### 2.7 Viability assay

CD146^+^ BMSCs from each subject were seeded in triplicate in 96-well plates at a density of 2.5 × 10^4^ cells per well. After 24 h, cells were treated with AMD3100, G-CSF, NE, or complete medium alone for 48 h at the concentrations described above. Cell metabolic activity was then assessed by MTT viability assay. Briefly, complete medium was replaced with vascular cell basal medium supplemented with 1% FBS for 24 h, followed by a PBS wash. A total of 100 µL of MTT solution (0.5 mg/mL) was added to each well and incubated for 3 h in the dark. Formazan crystals were solubilized by adding 150 µL per well of MTT eluent (4 mM HCl, 0.05% (v/v) Triton X-100 in isopropanol). After 20 min, absorbance was measured at 590 nm using a microplate reader.

CD34^+^ HSPC viability following 24 h treatment was evaluated by manual trypan cell count and expressed as the percentage of viable cells.

### 2.8 *In vitro* BM simplified experimental model to study CD146^+^ BMSCs-dependent CD34^+^ HSPC migration

**FIGURE 1:**
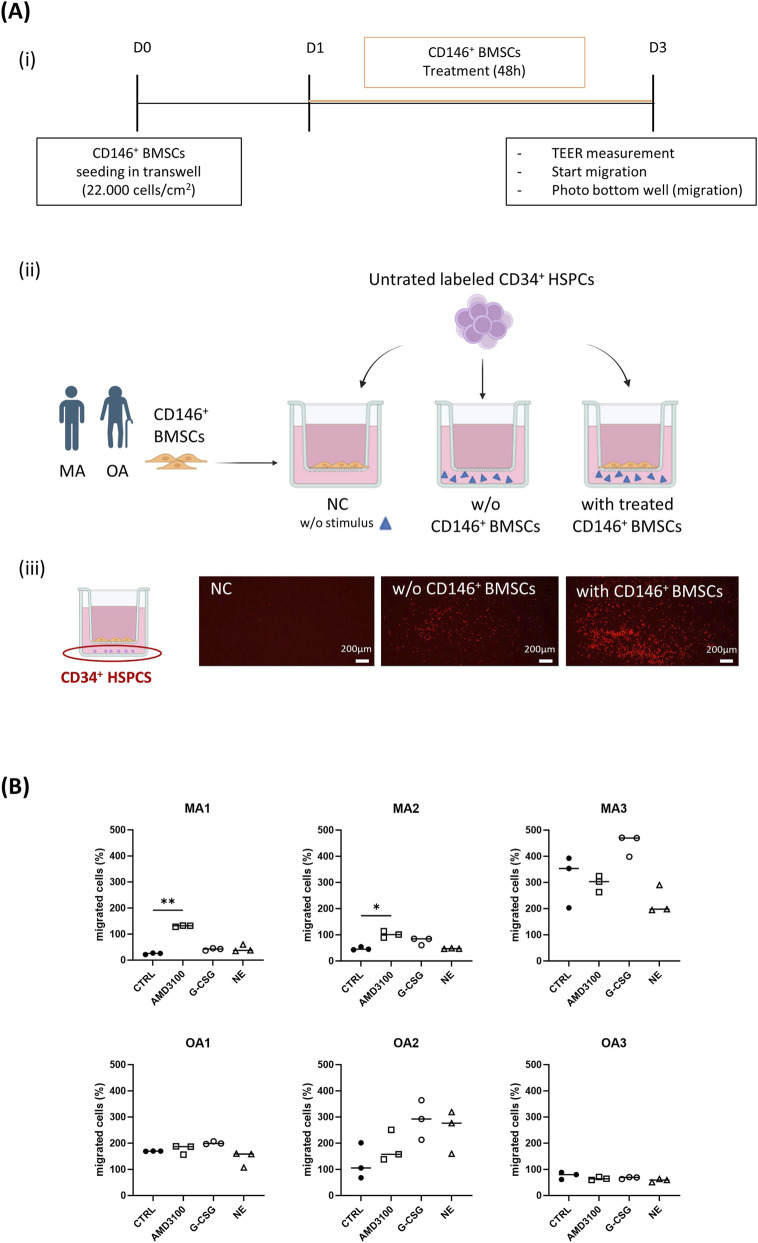
*In vitro* testing of the impact of age on human BMSC-mediated CD34^+^ HSPCs migration. **(A)** Schematic representation of the experimental setup, including (i) a timeline of the principal experimental steps, (ii) a graphic description of the different experimental conditions and (iii) a representative image showing migrated CD34^+^ HSPCs (red) in the different experimental conditions. **(B)** Age-dependent effects of mobilizing agents on CD146^+^ BMSC-mediated CD34^+^ HSPC transmigration; each graph represents an individual donor with its technical replicates, express as percentage of migrated cells relative to the internal reference (without CD146^+^ BMSCs). N = 3 subjects per group, with 3 technical replicates per subject. Statistical analysis was performed using the Kruskal–Wallis test. *p < 0.05; **p < 0.01. Negative control, NC; hematopoietic stem progenitor cells, HSPCs; bone marrow stromal cells, BMSCs; untreated control, CTRL; specific CXCR4 inhibitor, AMD3100; granulocyte colony-stimulating factor, G-CSF; norepinephrine, NE. (Panel A (ii) and (iii) created in https://BioRender.com).

As detailed in Figure 1A, CD146^+^ BMSCs were seeded at a density of 2.2 × 10^4^ cells/cm^2^ onto 24-well Transwell inserts with 8-µm pore size (Corning, cat # 3422). After 24 h, CD146^+^ BMSCs were treated with AMD3100, G-CSF, NE, or complete medium alone for 48 h. On the day of the migration assay, the integrity of the CD146^+^ BMSC layer was assessed by measuring transwell electrical resistance using an EVOM manual voltohmmeter (World Precision Instruments, model EVM-MT-023-01) according to the manufacturer’s instructions. Transepithelial/endothelial electrical resistance (TEER) was calculated by multiplying the measured resistance (Ω) by the membrane surface area (cm^2^).

CD146^+^ BMSCs were labeled with CellTracker™ Blue CMAC (Thermo Fisher Scientific, cat #C2110) at a final concentration of 25 μM, and CD34^+^ HSPCs were labeled with Calcein Red-Orange, AM (Life Technologies, cat #C34851) at 500 nM; both stainings were performed for 45 min at 37 °C. Labeled CD34^+^ HSPCs were washed and resuspended in vascular cell basal medium. Complete medium was used in the basal chamber as a chemoattractant, while vascular cell basal medium alone was used as a negative control (NC). As internal reference, CD34^+^ HSPC basal migration toward the chemoattractant was assessed in the absence of CD146^+^ BMSC layer without treatment with AMD3100, G-CSF, NE.

A total of 200 µL of vascular cell basal medium was added to the apical compartment, and 1 × 10^4^ CD34^+^ HSPCs in 50 µL were seeded onto each insert. Treatments were maintained throughout the migration assay. After 2 h, migration was stopped, and five images per replicate of the well bottom were acquired at ×4 magnification using a Leica DM IL Fluo microscope. Migrated cells were quantified using ImageJ software. Results were expressed as the percentage of migrated cells relative to the positive control condition, defined as migration in the absence of CD146^+^ BMSCs. Moreover, to assess the robustness of the BM simplified experimental model, the coefficient of variation (CV) was calculated for each sample under each condition across technical replicates, using the formula: standard deviation of the technical replicates divided by their mean, multiplied by 100.

### 2.9 RNA sequencing and transcriptome analysis

CD146^+^ BMSCs from each subject were plated at a density of 1.5 × 10^5^ cells per well in 6-well plates. After 3 days in culture the total RNA was isolated using the miRNeasy Kit (QIAGEN, Hilden, Germany; cat # 217084), including an on-column DNase digestion step. RNA was subsequently precipitated with ethanol to further improve purity.

Before library preparation, RNA samples were checked by evaluating quantity, quality and integrity. RNA concentration was determined using a Qubit Fluorometer (Life Technologies), RNA purity through NanoDrop 2000c spectrophotometer (Thermo Fisher Scientific) and integrity was assessed by using Agilent 4200 TapeStation system (Agilent Technologies). Sequencing libraries were generated from 400 ng of total RNA as input with the TruSeq Stranded Total RNA kit (Illumina), according to the manufacturer’s instruction. Libraries were sequenced at a final concentration of 1.3 pM in paired-end mode (2 × 100 bp) on a NovaSeq 6,000 platform (Illumina Inc.).

Raw sequencing reads were pre-processed as previously described (Melone et al., 2025).

Briefly, reads were demultiplexed using bcl2fastq (v2.20.0.422; Illumina, SCR-015058), and read quality was assessed employing FastQC (http://www.bioinformatics.babraham.ac.uk/projects/fastqc/). Adapter trimming was performed using cutadapt (v3.3) (Martin, 2011). Cleaned reads were aligned to the human reference genome (hg38) using STAR (v2.7.11b) (Dobin et al., 2013). Gene expression quantification was carried out with featureCounts (v2.0.1), using the GENCODE v46 primary assembly annotation. Transcripts supported by more than 10 raw counts were considered expressed.

Differential expression analysis was conducted in R using the DESeq2 package (v1.46.0) (Love et al., 2014) with default settings, retaining transcripts with an absolute fold change ≥1.5 and an adjusted p-value ≤0.05. Heatmap was created using the pheatmap library (v 1.0.13) on R using the log10 of the normalized counts and scaling by rows.

### 2.10 Bioinformatic analysis

STRING analysis was performed using a medium confidence interaction score (0.4), with a maximum of five interactions per protein on all the 9 genes differentially expressed. Gene Ontology (GO) biological process enrichment analysis was conducted using a false discovery rate (FDR) cutoff of 0.05.

### 2.11 Real-time polymerase chain reaction

CD146^+^ BMSCs (1.5 × 10^5^ cells per well) from each subject were seeded in 6-well plates. After 24 h, cells were treated for 48 h with mobilizing agents, after which total RNA was extracted using an RNA purification kit (miRNeasy Kit, QIAGEN, Hilden, Germany, cat # 217084) following the manufacturer’s instructions. Quantification was performed by using NanoDrop-1000 spectrophotometer and RNA quality was checked by OD 260-280 readings. The TaqMan® Reverse Transcription Reagents Kit (Thermo-Fisher) was used to reverse-transcribe 0.5 µg RNA, using Oligo d(T)_16_ in a final volume of 50 µL. Subsequently, 0.5 µL of the latter was used in each polymerase chain reaction. Real-time qPCR was performed with Sybr Green Master Mix in a final volume of 10 µL by the QuantStudio 6 flex (Applied Biosystems, Foster City, CA, United States of America) detection system. Data were obtained as Ct values, and the 2^−ΔΔCT^ method was used in the analysis, calculating the fold change of each transcript relative to the adult group mean or untreated samples and using histone 3 as reference gene. The specific primers used were: forward 5′-GTG​AAG​AAA​CCT​CAT​CGT​TAC​AGG​CCT​GGT-3′ and reverse 5′-CTG​CAA​AGC​ACC​AAT​AGC​TGC​ACT​CTG​GAA-3’ (for H3); forward 5′- AGG​TGG​ACG​CAC​ATG​ACT​GTG​A-3′ and reverse 5′-AGG​TTG​CAG​GCG​CGC​ATC​ATG-3′ (for TMEM204); forward 5′-CAG​GCT​GAA​GTC​CAG​ATA​GAG​C-3′ and 5′- GTG​CAT​GAT​CCT​GGT​TGT​TAG​GC-3’ (for NRK); forward 5′- ACT​GGA​CCG​AGC​TCA​CCA​ACT​G-3′ and reverse 5′-GTC​TCG​TAG​CAG​GGT​GAT​GTA​G-3’ (for HS6ST1); forward 5′-CAA​TGC​CGA​GTT​CTC​CTT​CCA​TG-3′ and reverse 5′- TGA​TGT​GCA​GGG​TGT​ATC​GGG​T-3’ (for ALOX15B); forward 5′- ATA​GGA​CCA​AGA​CCC​CTG​GA-3′ and reverse 5′-GGT​GAT​GTC​CTC​AAT​CTG​CCT-3’ (for ENSG00000213058); forward 5′- AGG​TGG​AGC​ATT​TCT​CGC​GCT​A-3′ and reverse 5′- GAA​TGT​TGG​CGA​GTC​TCA​CAG​G-3’ (for PDK4); forward 5′- GGG​TCG​CTT​TTG​GGA​TTA​CCT​G-3′ and reverse 5′- CAA​CTC​CTT​CAT​GGT​CTC​GTC​C-3’ (for APOE); forward 5′-TAT​GCG​TGC​TGG​CTA​CTA​CCG​A-3′ and reverse 5′- GGT​TAA​TCC​CAC​AGC​CAG​TGT​AG-3’ (for AQP1); forward 5′- GCG​CCT​CTA​CTA​CAA​ACT​GGG​C-3′ and reverse 5′- CTC​ATA​GGC​ACG​AAT​CCG​CTT​G-3’ (for LMO2); forward 5′-GAT​TCT​GTG​CCC​ACA​GTA​AGG​C-3′ and reverse 5′- TGG​TCA​CAG​AGC​CAC​CTT​CTT​G-3’ (for VCAM-1); forward 5′- CTC​AAC​ACT​CCA​AAC​TGT​GCC​C-3′ and reverse 5′-CTC​CAG​GTA​CTC​CTG​AAT​CCA​C-3’ (for CXCL12); forward 5′-ATG​AAG​CAG​CCC​AGA​TGT​GGA​G-3′ and reverse 5′- TGG​TCC​ACA​TCT​GCT​CTT​GGC​A-3’ (for MMP1); forward 5′- AGC​GAG​TGG​ATG​CCG​CCT​TTA​A-3′ and reverse 5′-CAT​TCC​AGG​CAT​CTG​CGA​TGA​G-3’ (for MMP2); forward 5′-CCA​GAA​GGA​ACA​GTG​GTT​TGG​C-3′ and reverse 3′- ACT​GTC​CTC​TGG​GCT​TGG​TGT​T-3’ (for CD44); forward 5′- AGC​CTG​CGA​AAG​CCT​TTT​GGT​G-3′ and reverse 5′-GGC​TTC​ACA​TTC​AGC​AAA​CCT​GG-3’ (for PPARG2); forward 5′-AGC​GGC​TGA​CGT​GTG​CAG​TAA​T-3′ and reverse 3′- TCT​GAG​ACC​TCT​GGC​TTC​GTC​A-3’ (for ICAM-1); forward 5′- CTG​GAG​ACT​CCA​GCC​TAC​ACT​G-3′ and reverse 5′-CTG​CCC​TTG​TAA​GAC​TTG​GCT​G-3’ (for KITLG); forward 5′-GGT​CAA​GCA​ACC​CAG​CCT​TTT​C-3′ and reverse 5′- CAG​GTC​ATT​CCA​GCA​GAG​CCA​A-3’ (for Tie2/TEK); forward 5′- CTG​CCA​AGT​GAT​TGG​TGC​TTC​TG -3′and reverse 5′-AAT​GGT​GCG​CTT​CGG​GTC​TGA​T -3’ (for PRX1); forward 5′-TCC​ACT​GCA​AGG​AAC​AAC​AG-3′ and reverse 5′- TAA​GCG​TGC​TCC​CAC​ACA​T-3’ (for SOD2); forward 5′-GGA​AGT​GGA​TGT​GGA​CAC​CAG​A-3′ and reverse 5′-GCT​TGT​AGT​CAG​GAT​GGT​TTG​CG-3’ (for GCLC). Data are expressed as mean ± standard deviation (SD), as indicated.

### 2.12 CCM-induced migration assay

Migration of PBMCs was performed using CIM-plate, a 16-well modified Boyden chamber composed of an upper and a lower chamber that snapped together to form a tight seal. The 3x10^5^ PBMCs isolated from patients (as described in the section above “Cells employed in functional assays”) were seeded in the upper chamber of the CIM-plate-16 in RPMI medium, and the CCM of treated (G-CSF, AMD3100, NE) or untreated MA/OA CD146^+^ BMSCs was added in the lower chamber. RPMI supplemented with 10% FBS was added as a chemoattractant in the lower chamber (positive control), while basal RPMI was added in the lower chamber as a negative control. Cell migration was monitored for up to 4 h. The migrated cells in the lower chamber were imaged using Leica optical microscope and quantified by staining them for 10 min with Hoechst 33,342 (0.2 μg/mL). The fluorescence was measured at 460 nm with an excitation wavelength of 345 nm.

### 2.13 ELISA assay

The supernatant was isolated from MA/OA CD146^+^ BMSCs exposed or not to G-CSF, AMD3100, or NE and used in ELISA assay. The amounts of the soluble proteins of interest - CXCL12, Vascular Cell Adhesion Molecule 1 (VCAM-1) and Intercellular Adhesion Molecule-1 (ICAM-1) - released by CD146^+^ BMSCs were measured using specific kits (#DSA00, #DY809-05, and #DY720, R&D Systems, Minneapolis, MN, United States of America), following the manufacturer’s instructions.

### 2.14 Reactive oxygen species (ROS) measurement

Confluent endothelial cells (ECs) incubated for 6 h with the CCM of treated (G-CSF, AMD3100, NE) or untreated MA/OA CD146^+^ BMSCs were assayed for intracellular ROS using 2′,7′-dichlorofluorescein diacetate (DCFH-DA). Briefly, the cells were incubated with 10 μM DCFH-DA (30 min at 37 °C), washed with PBS, and the DCF fluorescence emission was detected at 530 nm with an excitation wavelength of 495 nm in a 96-well microplate reader (GENios, Tecan). Immediately after DCF measurements, cells were further incubated for 10 min with Hoechst 33,342 (0.2 μg/mL) and the fluorescence was measured at 460 nm (with an excitation wavelength of 345 nm). ROS levels were expressed as DCF/Hoechst fluorescence units.

### 2.15 Metabolic analysis

Metabolic analyses were conducted in CD146^+^ BMSCs from middle-aged and older adults, either untreated or after 48 h treatment with AMD3100, G-CSF or NE. Mitochondrial respiration was measured through oxygen consumption rate (OCR; pmol/min) directly on CD146^+^ BMSCs within the Seahorse XFe24 Analyzer (Agilent, CA, United States of America). Assay preparation and procedure, as outlined by Agilent (Seahorse XF Cell Mito Stress Test Kit User Guide) were followed to carry out titrations and metabolic tests using Xfe 24-well. Sensor cartridges were hydrated using Seahorse XF Calibrant, pH 7.4 (Agilent), the day before assay, and placed in a 37 °C incubator overnight. On the day of the run, assay media were prepared according to Agilent protocols and placed in 37 °C incubator prior to use. After CD146^+^ BMSCs were counted and resuspended in assay media, they were seeded in the Seahorse cell culture plate in quadruplicate, at a density of 1 x 10^4^ cells. After the cells were seeded, the plate was allowed to rest in the tissue culture hood at room temperature for 60 min to minimize the cell growth edge effect, as recommended in the Agilent user guide.

The day of the assay, the cell culture growth medium in the cell culture microplate was changed to warmed assay medium using a multichannel pipette, and the cell culture microplate was placed into a 37 °C non-CO_2_ incubator for 45 min to 1 h before the assay. During this incubation period, 10 × concentrated drugs were loaded into their respective injection ports.

The sensor cartridge containing the study drugs was then inserted into the analyzer for calibration. Once the analyzer was calibrated, the calibration plate was replaced by the microplate containing the cells, and the assay protocol was initiated.

For CD146^+^ BMSCs, the respiratory stocks were loaded into the drug ports in the following order: (A) oligomycin (1.5 µM final), (B) FCCP (2.0 µM final), and (C) antimycin A + rotenone (0.5 µM final).

This sequential addition of specific electron transport chain modulators through the instrument’s injection ports interrogated mitochondrial oxidative phosphorylation. At first, oligomycin inhibited ATP synthase, revealing the proportion of basal respiration devoted to ATP production. Next, the uncoupler FCCP collapsed the proton gradient to elicit maximal respiration and determine spare respiratory capacity. Finally, rotenone and antimycin A (Rot/AA) blocked Complex I and III, respectively, to quantify nonmitochondrial oxygen consumption. From these perturbations, the assay yields quantitative parameters-basal respiration, ATP-linked respiration, proton leak, maximal respiration, spare capacity, and nonmitochondrial respiration-enabling detailed characterization of mitochondrial function and dysfunction in live cells.

### 2.16 Statistical analysis

Statistical analyses were performed using appropriate non-parametric tests. Comparisons between middle-aged adult (MA) and older adult (OA) groups were conducted using the Mann-Whitney U test. Unless otherwise specified, comparisons among multiple treatment conditions within the same group (MA or OA) were performed using the Kruskal–Wallis test. Data are displayed as individual data points with the median indicated. *p < 0.05, **p < 0.01 and ***p < 0.001 vs. control group.

## 3 Results

### 3.1 *In vitro* BM modelling allows the study of donor age-driven differences in CD146^+^BMSC-mediated migration of CD34^+^ HSPC

To investigate the contribution of perivascular BMSCs in CD34^+^ HSPC mobilization and the effect of mobilizing drugs, an *in vitro* BM simplified experimental model was developed culturing human CD146^+^ BMSCs in a transwell-based system. Before testing the mobilizing drugs in the *in vitro* co-culture system, their effect on CD146^+^ BMSCs and BM CD34^+^ HSPCs cell viability was investigated. The results showed that cell viability was not affected by the selected dosage of AMD3100, G-CSF, or NE drug treatments (Supplementary Figure S2).

Primary CD146^+^ BMSCs derived from randomly selected male samples from middle-aged (MA) or older adult (OA) subjects (N = 3) (Table 1) were seeded on the upper side of a permeable membrane. The BM simplified experimental model was initially characterized by assessing barrier integrity through TEER measurements, which revealed no differences in CD146^+^ BMSC layer permeability between control and mobilizer-treated conditions (Supplementary Figure S3). Model functionality was evaluated by measuring the directional migration of BM-derived CD34^+^ HSPCs across the CD146^+^ BMSC barrier in response to complete medium as chemotactic stimuli, expressed in percentage relative to migration in the absence of CD146^+^ BMSC barrier used as internal reference (Figure 1A). Our *in vitro* co-culture system exhibits a consistent coefficient of variation across technical replicates (16.77% ± 12.74, mean ± SD), indicating good reproducibility and robustness. Whereas CD34^+^ HSPC migration was highly reproducible across technical replicates within each subject, it displayed marked inter-subject variability, supporting the suitability of this system for applications in personalized medicine. Two of the three middle-aged adult subjects showed a significant increase in CD34^+^ HSPC migration in response to AMD3100 treatment, whereas the third middle-aged adult subject exhibited a non-significant trend toward increased migration under G-CSF treatment. Notably, the subject who did not respond to AMD3100 displayed a substantially elevated basal migratory activity (Ctrl, ∼350% migrated cells), compared with the other two middle-aged subjects, who exhibited fewer than 50% migrated cells under basal conditions. In the older adult group, conversely, only one of three subjects showed a non-significant trend toward increased migration in response to drug treatments (OA2). Interestingly, in a subset of subjects (MA3, OA1, OA2), CD34^+^ HSPC migration was higher in the presence of CD146^+^ BMSCs compared with conditions lacking CD146^+^ BMSCs, as highlighted by values exceeding 100% migrated cells, suggesting a donor-dependent effect of CD146^+^ BMSCs on CD34^+^ HSPC migration (Figure 1B). Overall, these data indicate that CD146^+^ BMSCs play a key role in regulating CD34^+^ HSPC migration and suggest that CD146^+^ BMSCs derived from aged subjects are less responsive to AMD3100 treatment.

### 3.2 Aging induces downregulation of *NRK* and upregulation of *PDK4*, *AQP1,* and *LMO2* in CD146^+^ BMSCs

**FIGURE 2:**
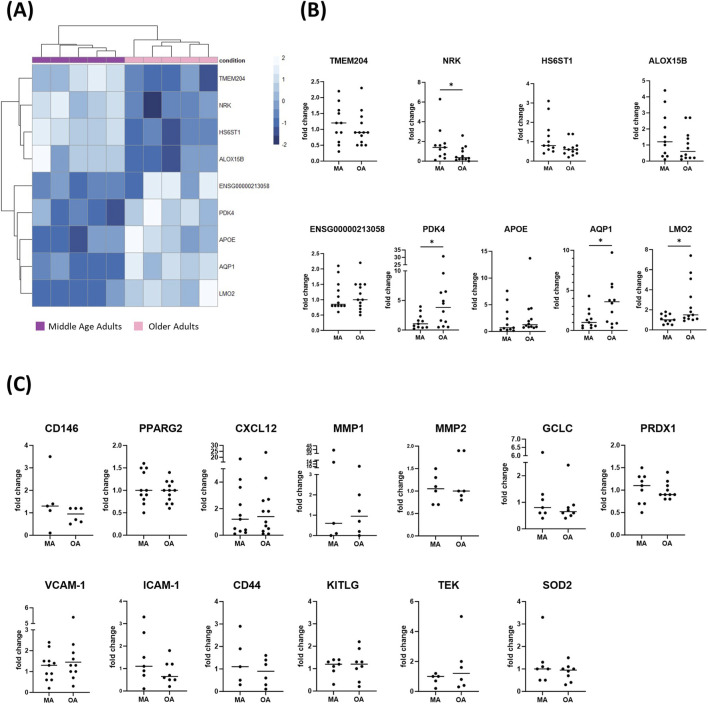
Transcriptomic analysis of CD146^+^ BMSCs from middle-aged adult and older adult subjects. **(A)** Heatmap of differentially expressed genes in CD146^+^ BM-MSCs from older adults compared with middle-aged adults, as identified by RNA-seq analysis. (N = 5). **(B)** Real Time PCR validation of *NRK, PDK4, AQP1*, and *LMO2* differential expression in CD146^+^ BMSCs from older adult (N = 12) and middle-aged adult (N = 11) subjects. **(C)** Expression of genes involved in cell migration, adhesion and redox regulation in CD146^+^ BMSCs derived from a subgroup of middle-aged adult versus older adult subjects (N_min_: 5, N_max_: 12). Statistical analysis was performed using the Mann-Whitney test. *p < 0.05.

Migration assay had shown that mobilizers had less or no impact on migration induced by CD146^+^ BMSCs isolated from older adult subjects (Figure 1B). In order to investigate whether this was due to a differential gene expression between middle-aged adult and older adult subjects, we conducted RNA-seq analysis on CD146^+^ BMSCs. RNA-seq analysis revealed a statistically significant downregulation of *TMEM204*, *NRK*, *HS6ST1,* and *ALOX15B*, and a statistically significant upregulation of *ENSG00000213058*, *PDK4*, *APOE*, *AQP1,* and *LMO2* in CD146^+^ BMSCs derived from older adult subjects compared with those from middle-aged adult donors (N = 5, of which 60% female), as highlighted in the heatmap of Figure 2A.

STRING analysis did not identify direct interactions among the nine RNA-seq-derived differentially expressed genes (DEGs) (Supplementary Figure S4A). However, gene ontology analysis of the top 30 DEGs ranked by unadjusted p-value indicated an enrichment of vascular-related genes (Supplementary Figure S4B). This is in line with the current literature highlighting the impact of aging on vascular function and angiogenesis (Ungvari et al., 2018), and the direct involvement of BMSCs in age-related impairment of vascular functional (Duscher et al., 2014).

Real Time PCR analysis using RNA from subjects with a balanced sex distribution (Table 1), validated the differential expression of *NRK*, *PDK4*, *AQP1,* and *LMO2* (MA: N = 11, OA: N = 12) (Figure 2B).

Moreover, we randomly selected a subset of subjects to extend the analysis to genes known to be involved in BM cell mobilization, including CAMs, redox homeostasis-related genes, proteases, and the chemokine CXCL12 (MA: N ranging from 5 to 11, OA: N ranging from 6 to 12; depending on the specific gene). In agreement with the RNAseq data that did not identify them among the DEGs, no statistically significant differences were observed in the relative expression of the selected genes when comparing CD146^+^ BMSCs derived from MA and OA subjects (Figure 2C).

### 3.3 AMD3100, G-CSF, and norepinephrine differentially regulate gene expression in CD146^+^ BMSCs in an age-dependent manner

**FIGURE 3:**
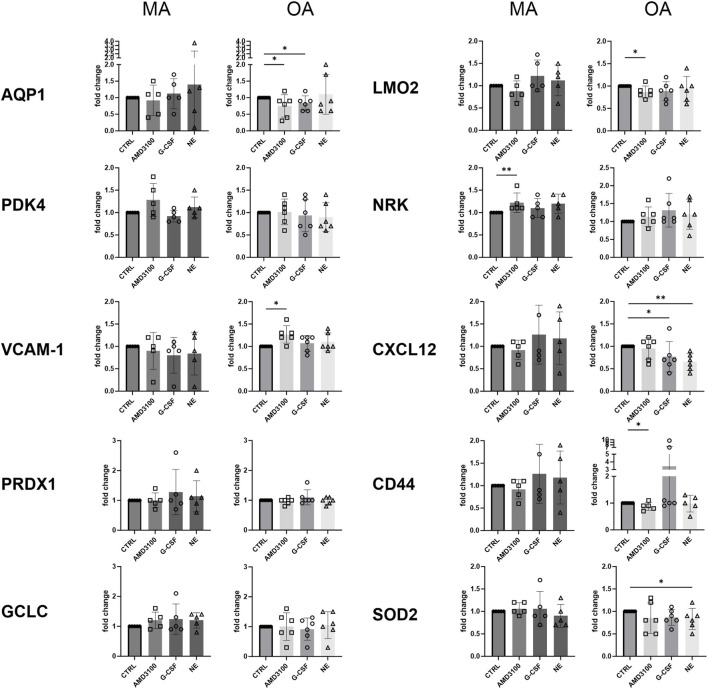
Treatment-induced modulation of gene expression in CD146^+^ BMSCs from middle-aged and older adults. Gene expression levels were analyzed in CD146^+^ BMSCs derived from adult and aged donors following treatment with the indicated agents. For each gene, separate graphs show middle-aged and older adults -derived cells, respectively, with each graph depicting the effects of the different treatments. Data are shown for N = 6 donors per group. Statistical analysis was performed using the Mann-Whitney test. *p < 0.05; **p < 0.01.

In order to identify the key molecular changes of CD146^+^ BMSCs associated with the differential migratory response of CD34^+^ HSPCs observed after treatment with AMD3100 (Figure 2B), we analyzed the gene expression of selected age-regulated, migration- and redox-related genes upon mobilizers’ treatment (Figure 3). Interestingly, as observed for the migration assay, the mobilizers-dependent impact, evaluated on the relative age-specific untreated control, was different between older and middle-aged adult subjects. In particular, treatment with AMD3100 provoked an increase of *NRK* expression in cells derived from middle MA, while in OA it resulted in increased *VCAM-1* expression and reduced expression of *AQP1*, *LMO2*, and *CD44* compared to untreated control cells. G-CSF treatment did not induce significant gene expression changes in MA cells but led to decreased *AQP1* and *CXCL12* expression in OA-derived CD146^+^ BMSCs. NE treatment did not affect gene expression in MA cells, whereas in OA cells it reduced *CXCL12* and *SOD2* expression.

AMD3100 produced the most pronounced gene regulatory effects among the mobilizing agents, and *NRK* upregulation in MA may underlie the increased of CD34^+^ HSPC transmigrated cells observed in its presence. Interestingly, under our experimental conditions, AMD3100 was the only mobilizer able to modulate CAMs expression (*VCAM-1* and *CD44*), while showing no effect on *CXCL12* expression. In contrast, but in line with the literature (Day et al., 2015; Lucas et al., 2008), both G-CSF and NE were able to reduce *CXCL12* expression in BMSC. Collectively, these findings support the notion that distinct mobilizers activate divergent signaling pathways in CD146^+^ BMSCs.

### 3.4 Mobilizing agents do not alter oxygen consumption rates in middle-aged and older adult CD146^+^ BMSCs

**FIGURE 4:**
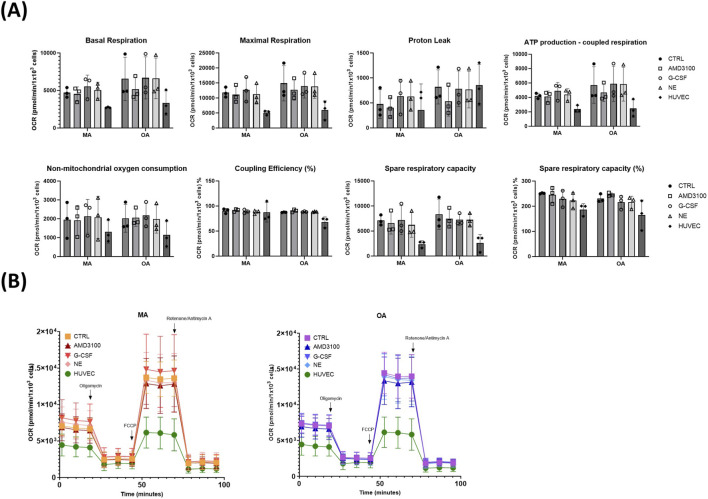
Mobilizing agent treatments do not alter oxidative respiration in middle-aged adult and older adult CD146^+^ BM-MSCs. **(A)** Bar charts summarize average OCR values for derived parameters of respiration during regions of metabolic interest (basal respiration, maximal respiration, proton leak, ATP production-coupled respiration, non-mitochondrial oxygen consumption, coupling efficiency percentage, spare respiratory capacity, spare respiratory capacity percentage). Data is presented as mean ± SD (N = 3 independent experiments, each performed with 4 technical replicates); **(B)** Mitochondrial respiration kinetic profile, showing oxygen consumption rate in middle-aged adult CD146^+^ BM-MSCs (left) and older adult (right), with the sequential injection of metabolic inhibitors at different time point, indicated by arrows. Colors designative of different treatment conditions (NT, AMD, G-CSF and NE) are indicated in the legend at the top left part of each plot. Green line is the same for both plots and corresponds to untreated HUVEC cells (used as internal control reference). Error bars represent standard deviation (N = 3 independent experiments, each performed with 4 technical replicates), Oxygen consumption rate, OCR.

To check whether CD146^+^ BMSCs could impact on CD34^+^ HSPCs cells, and thus their mobility, through altered metabolic activity, we used Seahorse XF technology to analyze oxidative phosphorylation in real time on the same samples used in the migration assay, in presence or absence of mobilizers (Figure 4). Oxygen consumption rate (OCR) was used as a measurement of oxidative phosphorylation and low-rate HUVEC cells were added to each assay run, as internal control reference.

Several parameters pertinent to mitochondrial respiration, including basal respiration, maximal respiration, proton leak and ATP production, showed a trend towards higher values in untreated OA subjects compared to untreated MA ones (see Figure 4, upper part of panel A, “CTRL” bars). Conversely, treatment with AMD3100, but not the other mobilizers (G-CSF or NE), seemed to impact on these parameters more in OA than MA subjects, driving a decrease towards “MA CTRL” values. However, the background oxygen use (non-mitochondrial oxygen consumption) was stable and the overall efficiency did not undergo any age- or treatment-dependent variation, with coupling efficiency and respiratory capacity being similar throughout the different conditions (see Figure 4, lower part of panel A).

Indeed, the overall kinetics graphs did not show any significant differences between MA and OA subjects or between untreated and mobilizer-treated conditions (Figure 4B), suggesting a physiological adaptation to potentially higher energy demands rather than a fundamental defect.

### 3.5 Impact of aging on the paracrine activity of CD146^+^ BMSCs

Given the age-dependent remodeling observed in multiple molecular pathways in CD146^+^ BMSCs, which did not appear to directly regulate HSPCs mobilization, and in the absence of overt metabolic involvement, we next investigated whether age could impact CD146^+^ BMSC paracrine activity, promoting a pro-inflammatory milieu. Thus, we investigated the composition and functional activity of CCM from middle-aged vs. older adult CD146^+^ BMSCs.

To test the chemoattractant activity on CD146^+^ BMSC-derived CCM, PBMCs were used for their greater accessibility and for the similarity in CXCL12 chemoattractant mechanism shared with BM-derived CD34^+^ (Meditz et al., 2008; Janssens et al., 2018).

**FIGURE 5:**
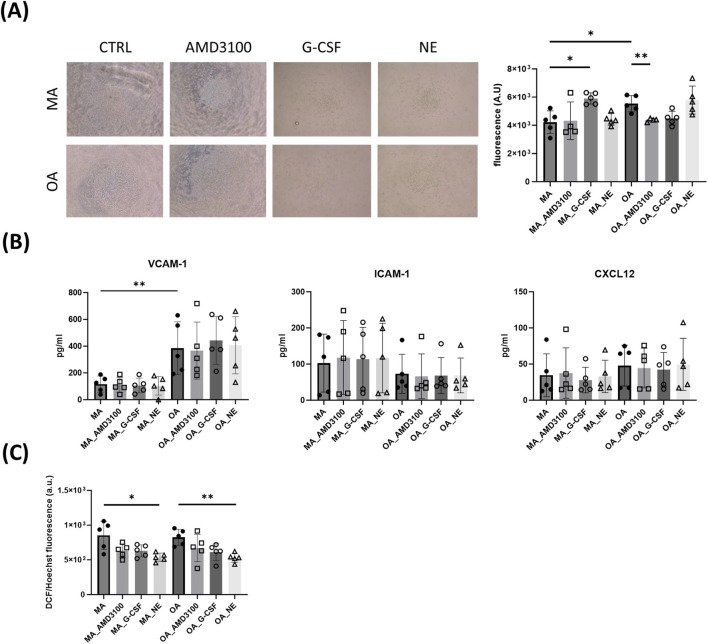
Increased chemoattractive potential of CD146^+^ BMSC-CCM derived from older donors compared to middle-aged adult. **(A)** PBMC migration assay toward CD146^+^ BMSC-CCM; **(B)** ELISA of CD146^+^ BMSC-CCM; **(C)** effects of CCM derived from mobilizing agent-treated CD146^+^ BM-MSCs on endothelial cell ROS levels. Data are shown for N = 5 donors per group. Statistical analysis was performed using the Mann-Whitney test for comparisons between adult and aged donors and the Kruskal–Wallis test for treatment effects. *p < 0.05; **p < 0.01. Cell conditioned medium, CCM; peripheral blood mononuclear cells, PBMCs; enzyme-linked immunosorbent assay, ELISA.

We found that CD146^+^ BMSC-CCM from older adults exhibited greater *in vitro* chemoattractant activity on PBMCs compared to CCM isolated from middle-aged adults (Figure 5A). Accordingly, ELISA assays showed that secreted VCAM-1 was increased in the CCM of older adult CD146^+^ BMSCs, in comparison to middle-aged adult ones (Figure 5B). Whereas no difference was observed in CXCL12 and ICAM-1 content among CCM from all conditions.

Mobilizing agents differentially affected the chemoattractant activity of CD146^+^ BMSC-CCM in MA and OA groups. Specifically, CCM derived from AMD3100-treated older CD146^+^ BMSCs significantly reduced PBMC migration, whereas no effect was observed in CCM from treated MA-derived cells. On the other hand, G-CSF treatment increased PBMC migration only through CCM derived from the MA group (Figure 5A).

To further investigate the paracrine effects of factors released by CD146^+^ BMSCs, we measured intracellular ROS levels in EA.hy926 endothelial cells using DCF fluorescence following incubation with CCM obtained from treated (G-CSF, AMD3100, or NE) or untreated CD146^+^ BMSCs derived from MA and OA. Although no significant differences in endothelial ROS levels were observed following exposure to CCM from MA versus OA CD146^+^ BMSCs, CCM derived from NE-treated CD146^+^ BMSCs significantly reduced ROS levels in endothelial cells in an age-independent manner (Figure 5C). While NE has previously been reported to increase ROS levels in stem cells, acting as a key regulator of HSPC trafficking and proliferation (Golan et al., 2019; Golan et al., 2018), our findings reveal an additional beneficial effect of NE treatment mediated through the stromal compartment, resulting in reduced oxidative stress in endothelial cells. Surprisingly, the NE treatment did not reduce CXCL12 levels in CD146^+^ BMSC-CCM.

## 4 Discussion

Aging profoundly affects bone marrow structure and function and is accompanied by chronic low-grade inflammation, known as inflammaging, which disrupts hematopoietic homeostasis, negatively affecting cardiovascular health (Mangialardi et al., 2014; Bonora et al., 2022; Kohler et al., 2009; Aranda et al., 2025). Consistent with this, the frequency in the circulation of the BM-derived cardiovascular protective CD34^+^ HSPCs declines with age, and this reduction is associated with worse cardiovascular outcomes mobilization (Moresi et al., 2005; Shimizu et al., 2020; Cohen et al., 2013; Patel et al., 2015; Albiero and Fadini, 2018). These observations underscore the clinical relevance of CD34^+^ HSPC mobilization and highlight the need for a better understanding of the age-dependent mechanisms limiting mobilization efficiency.

Within the BM microenvironment, the perivascular niche plays a central role in regulating HSPCs’ retention and egress. Perivascular BMSCs are key niche components, providing structural support and paracrine signals that control CD34^+^ HSPC behavior (Albiero and Fadini, 2018; Itkin et al., 2016; Chang et al., 2022; Campanile et al., 2023).

Although aging is known to impair BMSC function, promoting cellular senescence, inflammatory signaling, and redox dysregulations, while altering niche-supportive capacity (Gnani et al., 2019; Stenderup et al., 2003; Stolzing et al., 2008; Zhou et al., 2008; Peffers et al., 2016; Mueller and Glowacki, 2001), the specific contribution of human perivascular BMSCs to age-related defects in CD34^+^ mobilization has remained poorly defined, partially due to the lack of physiologically relevant human-based *in vitro* models.

To date, much of our understanding of CD34^+^ HSPC mobilization derives from the study of diabetic mouse models, which exhibit impaired mobilization in response to mobilizing agents (a phenomenon commonly referred to as “diabetic mobilopathy”) (Albiero and Fadini, 2018). This heavy dependence on disease-specific animal models underscores a clear unmet need for human-derived, physiologically relevant *in vitro* systems that enable controlled, quantitative investigation of CD34^+^ HSPC mobilization while capturing age-dependent perivascular stromal regulation.

Early transwell-based assays enabled quantitative analysis of CD34^+^ migration and identified key chemokines and adhesion molecules involved in mobilization; however, these models frequently employed stromal or endothelial cells of murine source, or lacked the human stromal component altogether, limiting their translational relevance (Campanile et al., 2023; de Barros et al., 2010; Mohle et al., 1997; Aleman et al., 2019; Voermans et al., 2001; Kessinger et al., 2005; Kim and Broxmeyer, 1998; van Buul et al., 2002; Mohle et al., 1999; Aiuti et al., 1997). The development of more complex three-dimensional spheroid models using human cells has improved the representation of stromal architecture and cell-cell interactions, but it was not specifically designed to study directed CD34^+^ HSPC egress toward the circulation (de Barros et al., 2010). Another advanced *in vitro* model, which introduced perfusable endothelium and spatially defined niches within a microfluidic system, has primarily focused on stem cell maintenance or differentiation rather than quantitative migration (Aleman et al., 2019).

To address this gap, in a pilot study we designed a new *in vitro* human BM co-culture system, developed throughout the successful isolation and culture of primary human CD146^+^ BMSCs from two groups of patients of different ages that were used to test how aging influences the migration of CD34^+^ HSPCs in response to clinically relevant mobilizers (i.e., AMD3100, G-CSF, and NE).

An interesting and unexpected finding was the increased migration of CD34^+^ HSPCs observed in the BM simplified experimental model employing CD146^+^ BMSCs derived from OA donors compared to MA donors under untreated conditions. This result may suggest a potential compensatory mechanism; however, alternative explanations such as dysfunctional cellular senescence or biological variability cannot be excluded and need to be addressed in future studies.

Our findings suggest that the migratory response of CD34^+^ HSPCs to the CXCR4 antagonist AMD3100 may vary between individuals and appears to be influenced by age, with a reduced responsiveness observed in the older group. This result is consistent with clinical and experimental evidence showing heterogeneous mobilization efficiency, particularly in older individuals (Voermans et al., 2001; Stiff, 1999; Fadini and Avogaro, 2013). Notably, the use of this human simplified experimental model - which enables direct assessment of CD146^+^ BMSC-CD34^+^ HSPC interactions - suggests that aging may play an important role in shaping the contribution of perivascular stromal cells to CD34^+^ HSPC mobilization.

To further characterize the stromal contribution in BM HSPC mobilization, we conducted an extensive molecular analysis of the aging and mobilizer-associated gene expression. CD146^+^ BMSCs transcriptomic profiling revealed that the age of the patients is accompanied by a specific signature, despite preserved expression of canonical niche markers and retention factors. This result is in line with extensive literature reporting age-related stromal cell reprogramming (Gnani et al., 2019; Stenderup et al., 2003; Stolzing et al., 2008; Zhou et al., 2008; Peffers et al., 2016; Mueller and Glowacki, 2001). In particular, differential expression of *NRK*, *AQP1*, and *PDK4* is noteworthy, as these genes have been potentially implicated in the regulation of inflammation, cellular metabolism, migration, and adaptive responses to environmental cues, all of which are critical for niche function (He et al., 2024; Zannetti et al., 2020; Qin et al., 2020; Nakano et al., 2003; Khan et al., 2020; Jiang et al., 2025; Meng et al., 2014; Lee et al., 2022).

The Nik-related kinase, encoded by the *NRK* gene, is a serine/threonine kinase involved in cellular stress responses and tissue homeostasis, and it was the only gene we found significantly downregulated in older adult CD146^+^ BMSCs. Reduced *NRK* expression in arterial vascular smooth muscle cells has been associated with diabetes and cardiovascular disease in atherosclerotic patients (Lu et al., 2020), suggesting that *NRK* may contribute to the suppression of inflammatory signaling. In CD146^+^ BMSCs, this function could support the more efficient migratory response observed in the middle-aged group, although the underlying mechanisms regulating stromal-hematopoietic interactions remain to be clarified.

Another gene showing age-dependent modulation in CD146^+^ BMSCs in response to mobilizing agents is *AQP1*, an aquaporin isoform highly expressed in vascular endothelium and involved in the transport of both water and H_2_O_2_ across cellular membranes. In this case, we observed a significant upregulation of *AQP1* in older adults, in line with reports showing increased AQP1 distribution in aortic endothelial cells during aging in mice (Shabanian et al., 2024). Consistently, pharmacological AQP1 inhibition has been proposed as a strategy to prevent endothelial senescence and enhance angiogenic capacity in older individuals at increased risk of ischemic vascular disease (Shabanian et al., 2024). Together, these findings suggest that AQP1 may represent a relevant target for preserving CD146^+^ BMSC homeostasis during aging.

Moreover, altered expression of the oncogene *LMO2* supports a link between aging-related stromal changes and pathways involved in hematopoiesis and angiogenesis (Chambers and Rabbitts, 2015; Chen et al., 2021). In fact, *LMO2*-overexpressing human fibroblasts enhance the viability and proliferation of prostate cancer cells (Chen et al., 2021), demonstrating its capability to induce paracrine modulation.

The Pyruvate dehydrogenase kinase 4 (PDK4) is instead a key regulator of inflammation-associated metabolic reprogramming, promoting a shift from oxidative phosphorylation to glycolysis, and is known to be upregulated in patients with type 2 diabetes as well as in animal and human models of high-fat diet-induced metabolic stress (Lee, 2014). Therefore, the elevated expression of *PDK4* in CD146^+^ BMSCs from OA supported the inflammation signature associated with aging. Notably, despite increased *PDK4* expression, we did not observe major age-dependent metabolic alterations, suggesting that *PDK4* may exert context-specific, non-canonical roles in aging CD146^+^ BMSCs. It should be noted that the lack of statistical significance might be attributable to the limited sample size of the metabolic activity assay; therefore, larger studies are required to confirm these observations.

When we then analyzed the gene expression of several canonical niche-associated factors, including the perivascular niche marker *CD146*, the adipogenic differentiation regulator *PPARG2*, the chemokine *CXCL12*, the metalloproteinases *MMP1* and *MMP2*, or some CAM encoding genes (like *ICAM-1*, *VCAM-1*, and *CD44*), we did not observe any significant age-related changes. These results suggest that age does not uniformly disrupt canonical niche identity, but rather selectively alters specific regulatory pathways that may influence functional outcomes.

Furthermore, to better enquire age-effect on mobilizing mechanisms, we exposed CD146^+^ BMSCs from MA and OA subjects to mobilizing agents and measured the transcriptional levels of selected genes. This analysis revealed that, as expected, MA and OA exhibited distinct molecular responses to mobilizing agents, but surprisingly, OA showed a pronounced transcriptional change across *CXCL12*, CAMs, and oxidative stress-related genes. These broader molecular responses observed in older-aged stromal cells were not accompanied by improved CD34^+^ HSPC migration. In contrast, MA-derived niches exhibited more efficient functional responses despite more limited transcriptional modulation. This dissociation between molecular activation and functional competence may reflect compensatory or stress-related transcriptional programs that fail to restore effective niche function.

Interestingly, among the age-related differentially expressed genes, *AQP1*, *LMO2*, and *NRK* were also differentially regulated in response to mobilizing agents between study groups. Notably, AMD3100 induced the most pronounced gene regulation, and the upregulation of *NRK* in MA may contribute to the observed increased CD34^+^ HSPC migration, although this remains to be experimentally confirmed.

Additionally, AMD3100 was the only mobilizer able to modulate CAMs expression (*VCAM-1* and *CD44*), while showing no effect on *CXCL12* expression. In contrast, but in line with the literature (Day et al., 2015; Lucas et al., 2008), both G-CSF and NE were able to reduce *CXCL12* expression in BMSC. Collectively, these findings support the notion that distinct mobilizers activate divergent signaling pathways in CD146^+^ BMSCs.

Although not associated with functional migration outcomes, the downregulation of AQP1 induced by AMD3100 or G-CSF in CD146^+^ BMSCs from OA subjects, together with the decrease in *LMO2* following AMD3100 exposure, reinforces the hypothesis that AQP1 and LMO2 may represent relevant targets for preserving CD146^+^ BMSC homeostasis during aging.

Analysis of paracrine signaling provided further insight into age-dependent stromal regulation of CD34^+^ HSPC migration. Aged CD146^+^ BMSCs-derived CCM displayed a higher basal chemoattractant capacity toward PBMCs, which indicates a higher retention capacity associated with age rather than increased HSPC mobilization.

It has to be acknowledged that, although BM CD34^+^ HSPCs and PBMCs share some trafficking mechanisms, they are regulated by distinct, specialized chemoattractant gradients that regulate their retention in the BM versus migration to peripheral tissues. HSPC retention within the BM niche is primarily governed by the CXCL12/CXCR4 axis (Albiero and Fadini, 2018; Chang et al., 2022; Day et al., 2015). In contrast, PBMC migration to sites of inflammation depends on a broader repertoire of chemokine receptors (Wain et al., 2002). In this context, the enhanced chemoattractant activity observed in CCM from CD146^+^ BMSCs of older subjects is more likely indicative of an increased pro-inflammatory state rather than a direct effect on HSPC mobilization.

This interpretation is supported by significantly increased levels of secreted VCAM-1, a CAM expressed only in activated cells (Cannistra et al., 1994), corroborating a model in which aging reshapes the paracrine function of CD146^+^ BMSCs to favor inflammation and HSPC retention.

Nevertheless, the association between VCAM-1 levels and chemoattractant ability does not extend to pharmacological treatment effects. Despite treatment-dependent changes in migratory responses, VCAM-1 levels in conditioned media did not increase accordingly, indicating that VCAM-1 alone is not sufficient to account for the enhanced chemoattractant effects observed following mobilizing agent exposure. These findings imply that additional paracrine mechanisms, beyond VCAM-1, are engaged in response to mobilizing cues and contribute to the regulation of CD34^+^ HSPC migration in an age-dependent manner.

Given its central role in CD34^+^ HSPC mobilization, CXCL12 warrants particular attention. Several studies have claimed that CXCL12 levels decrease with aging in the human BM (Periyasamy-Thandavan et al., 2019; Gilbert et al., 2019); however, direct experimental evidence supporting this notion remains limited. On the other hand, reduced *CXCL12* expression in *in vitro* cultured murine BMSCs (Choudhery et al., 2012; Guang et al., 2013) is well established.

In our study, no significant differences in CXCL12 expression were observed between CD146^+^ BMSCs derived from MA and OA, which may reflect the high inter-individual variability of CXCL12 expression within this stromal population. The reduction in CXCL12 expression observed following G-CSF or NE treatment is consistent with previous reports linking these mobilizing agents to disruption of the BM chemotactic gradient (Day et al., 2015; Lucas et al., 2008). However, this reduction was not paralleled by a corresponding decrease at the protein level in secretome analyses. Given the high variability in secreted CXCL12 levels and the limited sample size analyzed, additional studies with larger cohorts will be required to determine whether changes in *CXCL12* transcription are uncoupled from CXCL12 secretion.

It is important to take into account that BM CD34^+^ egression, as well as their retention, is finely tuned by the recall of these cells to the perivascular niche and the chemoattractant gradient competition between the BM vascular niche and BM bloodstream (Albiero and Fadini, 2018).

In addition to chemokine signaling, reactive oxygen species emerged as an important paracrine regulator of CD34^+^ cell migration and retention (Itkin et al., 2016; Tesio et al., 2011; Golan et al., 2012). Therefore, we investigated endothelial ROS levels as a complementary readout of the paracrine effects exerted by CD146^+^ BMSCs. Of note, conditioned media derived from NE-treated CD146^+^ BMSCs significantly reduced DCF-detectable ROS levels in endothelial cells, irrespective of subject age. This finding suggests that NE stimulation induces a secretory profile capable of modulating the redox state of niche-associated endothelial cells, potentially promoting a more protective or quiescence-supportive microenvironment (Golan et al., 2019; Golan et al., 2018). In contrast to mobilizing agents such as G-CSF or AMD3100, which are commonly linked to oxidative stress and enhanced BM CD34^+^ egress, the antioxidant effect observed on endothelial cells subjected to NE-treated CCMs may reflect a distinct regulatory mechanism influencing endothelial-HSPC crosstalk. Importantly, the age-independent nature of this response indicates that NE-mediated redox modulation is preserved in MA and OA CD146^+^ BMSCs, supporting its potential relevance in aged BM niches where dysregulated ROS signaling is often implicated in impaired hematopoietic function.

The absence of age- or treatment-dependent differences in CXCL12 and ICAM-1 secretion, as well as in cellular respiration of CD146^+^ BMSCs, indicates that neither these fundamental BM molecules nor global metabolic activity are primary drivers of the observed functional differences. Together, these findings support a model in which CD146^+^ perivascular BMSCs regulate CD34^+^ HSPC migration through age-dependent, multifactorial mechanisms, involving selective transcriptional reprogramming and paracrine signaling rather than uniform alterations of classical niche factors.

Despite limitations related to the relatively small cohort size, the lack of subject-matched clinical mobilization data, and the fact that experiments were carried out under normoxic conditions rather than the hypoxic conditions, characteristic of the BM vascular niche (Kubota et al., 2008; Parmar et al., 2007), this study stands out as one of the few *in vitro* systems incorporating primary human cells, directly derived from the target tissue.

Having as a primary objective the establishment and validation of an *in vitro* BM simplified experimental model, rather than the comprehensive demonstration of an underlying biological mechanism or the derivation of broadly generalizable conclusions, the use of a limited but well-characterized set of primary samples is appropriate to demonstrate the feasibility, reproducibility, and potential applicability of the system. Such systems represent an important foundational step for translational biomedical research and provide a promising basis for future investigations, which will aim to expand the cohort and further validate the model in more diverse and representative conditions, such as more predictive and human-relevant drug screenings.

## Data Availability

The data presented in the study are deposited in the https://www.ebi.ac.uk/arrayexpress/ repository, accession number E-MTAB-16622.
