# Supplementary figures and images for: Impact of aging on CD146+ mesenchymal stromal cells-mediated regulation of bone marrow CD34+ hematopoietic stem/progenitor cell mobilization
Source: Front Bioeng Biotechnol. 2026 May 7;14:1802093. doi: 10.3389/fbioe.2026.1802093 (PMC13189962; doi:10.3389/fbioe.2026.1802093)

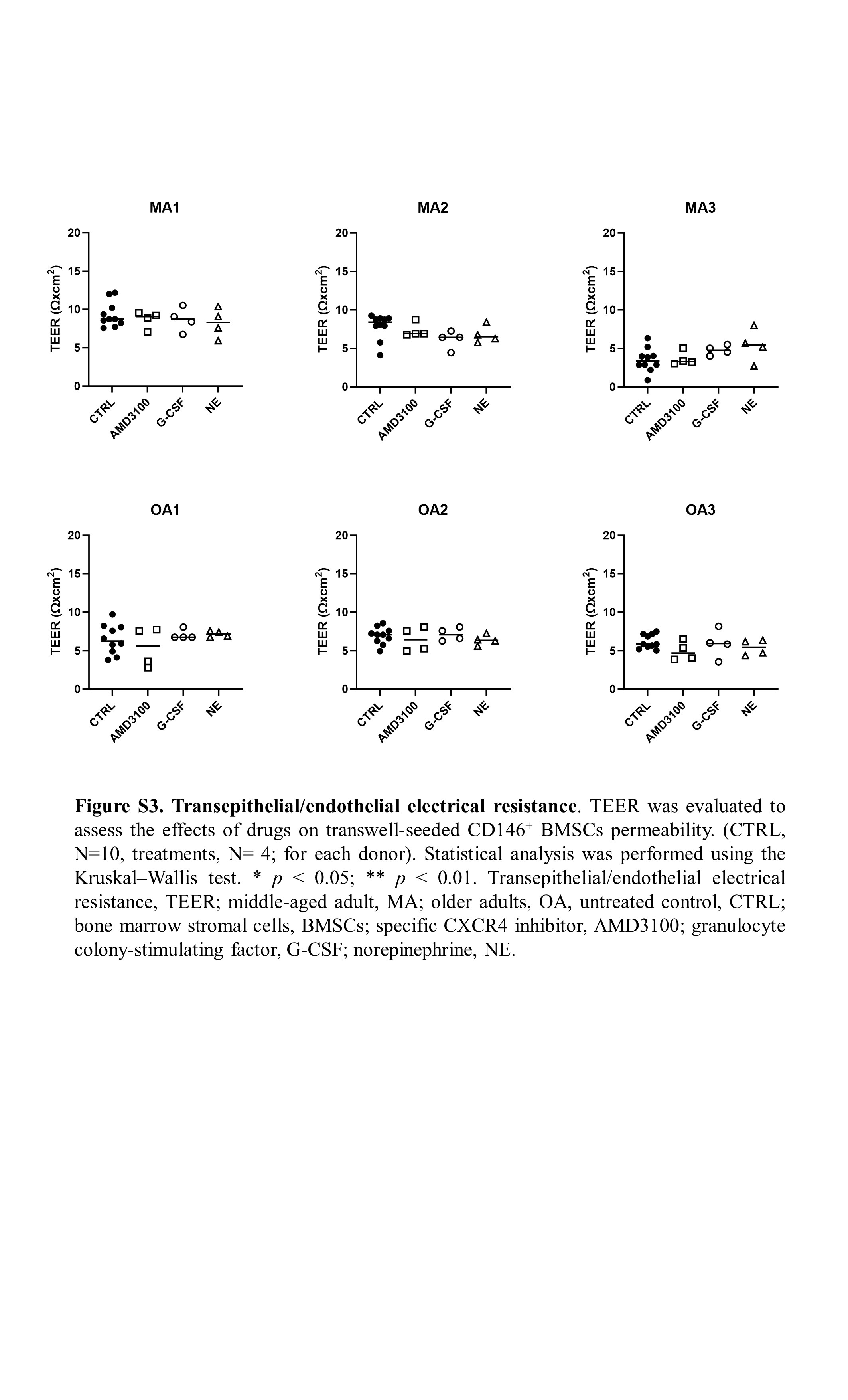

Supplement: Supplementary file 1 [file Image3.jpeg]

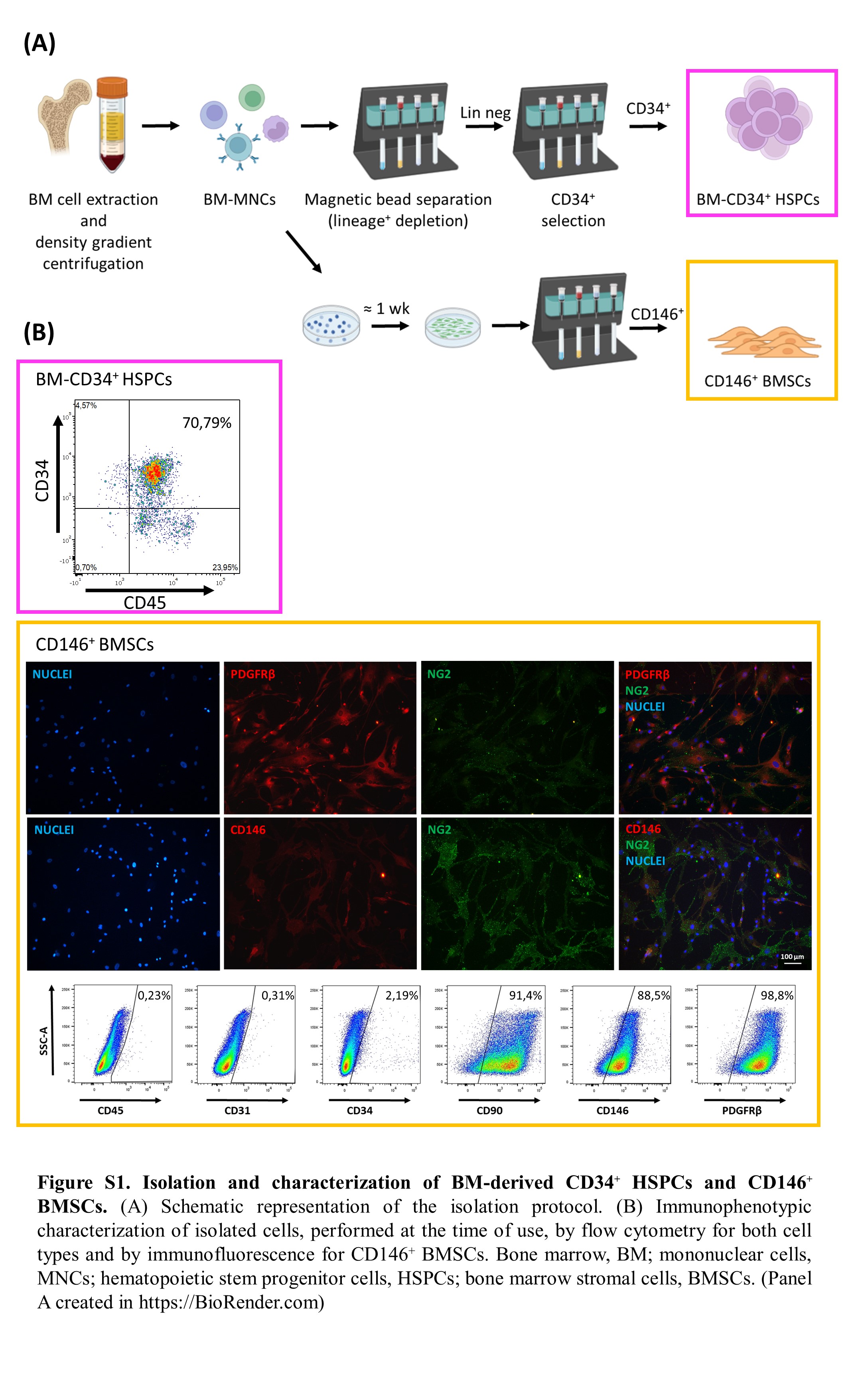

Supplement: Supplementary file 2 [file Image1.jpeg]

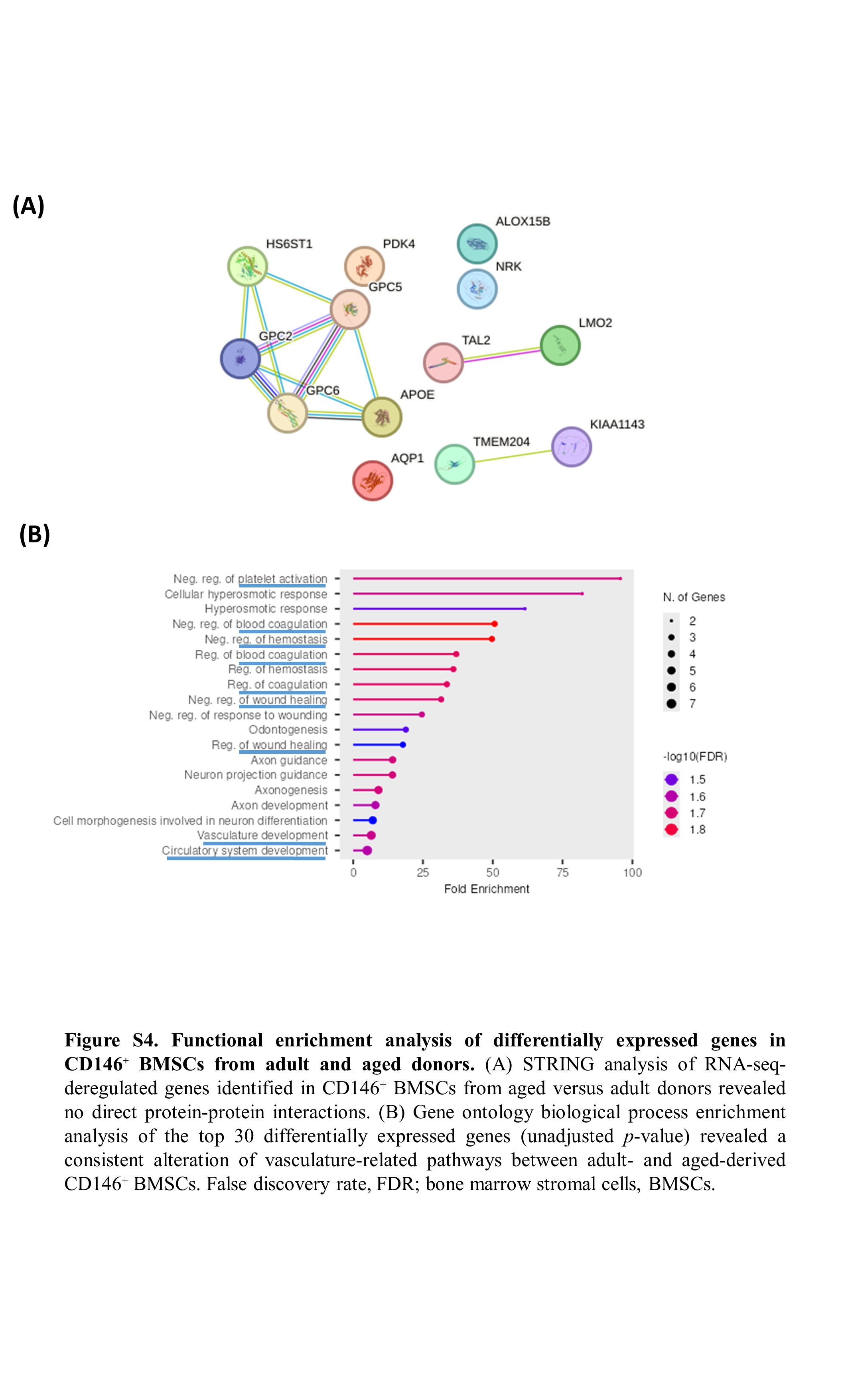

Supplement: Supplementary file 3 [file Image4.jpeg]

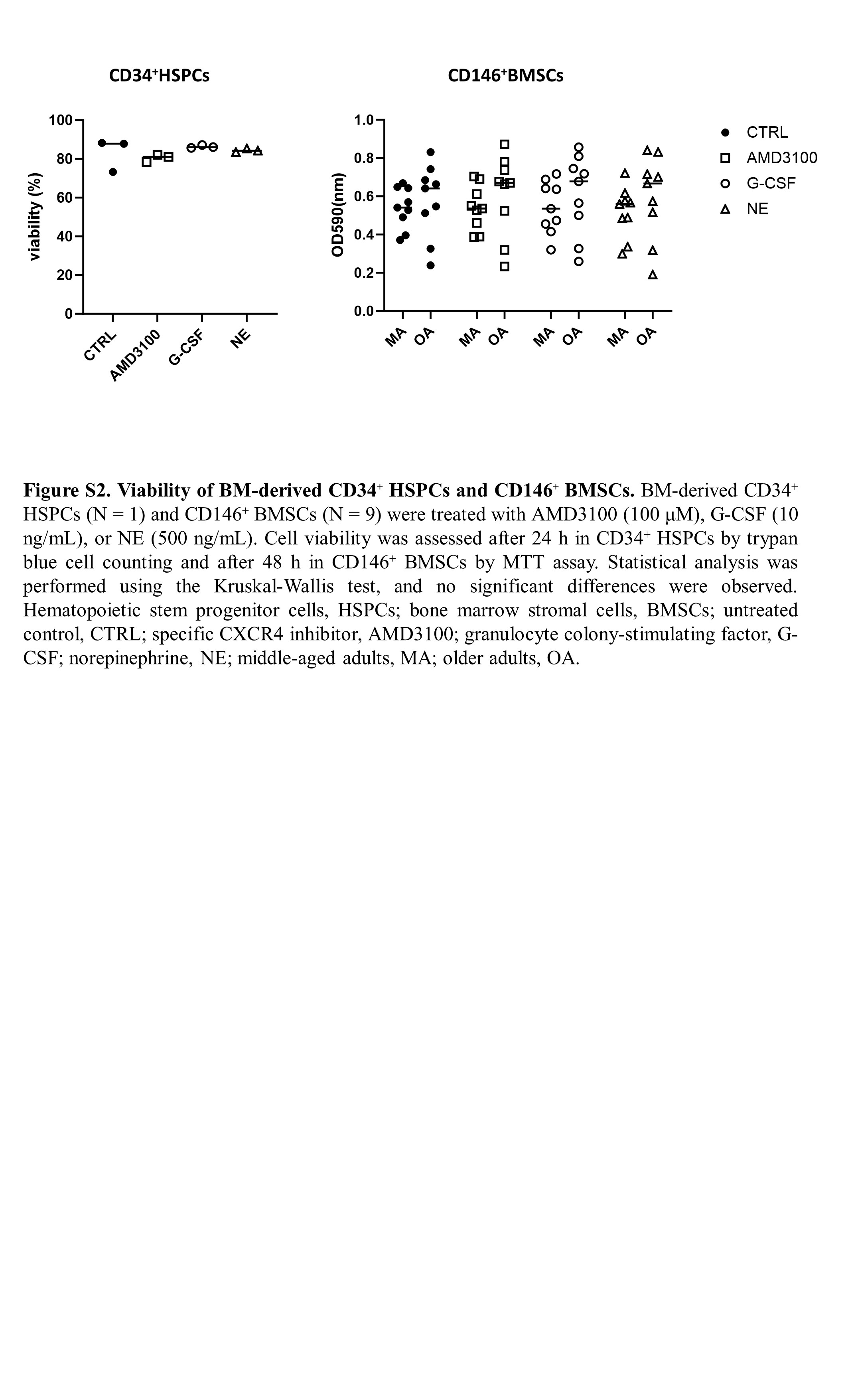

Supplement: Supplementary file 4 [file Image2.jpeg]
